# Diagnostic Accuracy of Computed Tomography for Radiographically Occult Scaphoid Fractures and Concordance With Magnetic Resonance Imaging: A Systematic Review

**DOI:** 10.7759/cureus.118468

**Published:** 2026-10-23

**Authors:** Rohin Wong, Andrien Rajakumar, Arkar Lwin, Zina Al-Kaisy, Paul Defty-Wallace, Rashed Al-Khudairi

**Affiliations:** 1 Emergency, Broomfield Hospital, Chelmsford, GBR; 2 Radiology, Broomfield Hospital, Chelmsford, GBR; 3 Intensive Care Unit, Broomfield Hospital, Chelmsford, GBR; 4 Radiology, Southend University Hospital NHS Foundation Trust, Southend-on-Sea, GBR; 5 Medical Education, Peterborough City Hospital, Peterborough, GBR

**Keywords:** computed tomography (ct ), diagnostic test accuracy, magnetic resonance (mr), occult fracture, scaphoid fracture

## Abstract

Scaphoid fractures are the most common carpal bone fracture. Non-union and avascular necrosis are complications of a missed fracture. Therefore, if plain radiographs are normal and symptoms persist, further imaging such as computed tomography (CT) or magnetic resonance imaging (MRI) are often performed. Previous systematic reviews have largely compared their diagnostic accuracy indirectly using separate patient cohorts. Our objective is to determine the diagnostic accuracy of CT for occult scaphoid fractures in patients who underwent both CT and MRI following a negative or equivocal initial radiograph, and to assess concordance between CT and MRI findings.

A systematic review was conducted in accordance with the Preferred Reporting Items for Systematic Reviews and Meta-Analyses (PRISMA) guidelines. OVID MEDLINE, OVID Embase, and the Cochrane Library were searched for studies published from inception up to August 31, 2026. Eligible studies enrolled adults with clinically suspected scaphoid fracture and a negative or equivocal initial radiograph who subsequently underwent both CT and MRI. Risk of bias was assessed using the Quality Assessment of Diagnostic Accuracy Studies 2 (QUADAS-2). Studies affected by partial verification or incorporation bias were excluded from quantitative synthesis. CT sensitivity and specificity were pooled using a bivariate random-effects model. Concordance between CT and MRI was reported using percentage agreement and Cohen’s kappa.

Eight studies met the inclusion criteria. Six studies contributed to the primary pooled analysis of CT diagnostic accuracy, with one study reporting two CT readers. Using the first reader data from that study, alongside the five other studies, pooled CT sensitivity was 79% (95% CI 61.5-89.8%) and specificity was 94.4% (95% CI 82.1-98.4%). A sensitivity analysis using the second reader produced a sensitivity of 69.2% (95% CI 48.0-84.6%), with an unchanged specificity of 94.4% (95% CI 85.5-98.0%). Three studies provided CT-MRI concordance data; two demonstrated almost perfect agreement, with Cohen’s kappa values of 0.91. The dual-energy CT study demonstrated lower concordance. Three studies used a reference standard independent of both CT and MRI and demonstrated mixed comparative findings. Overall, one study was judged to have low risk of bias, and seven were high risk of bias.

In the included studies, CT demonstrated high specificity but more limited sensitivity for occult scaphoid fracture in paired CT-MRI cohorts. These findings align with existing literature reviewing unpaired data sets. Therefore, the findings suggest that CT remains a relevant alternative to MRI, especially in centres where MRI is unavailable or contraindicated, or for surgical planning.

## Introduction and background

Scaphoid fractures are the most common carpal bone fracture, with an estimated incidence of 12.4 per 100,000 per year in the United Kingdom (UK) [1]. They typically result from a fall onto an outstretched hand [2,3].

The scaphoid is the largest bone of the proximal carpal row and comprises a proximal and distal pole separated centrally by its waist, which is the most common fracture site [1,4]. It receives a retrograde blood supply predominantly via volar and dorsal branches of the radial artery, which enter at the distal pole, as can be seen in Figure 1. As a result, scaphoid waist and proximal pole fractures are vulnerable to avascular necrosis if left untreated [5,6]. A missed fracture carries a risk of non-union, malunion, and avascular necrosis and may progress to chronic pain and scaphoid non-union advanced collapse [7-10].

**Figure 1:**
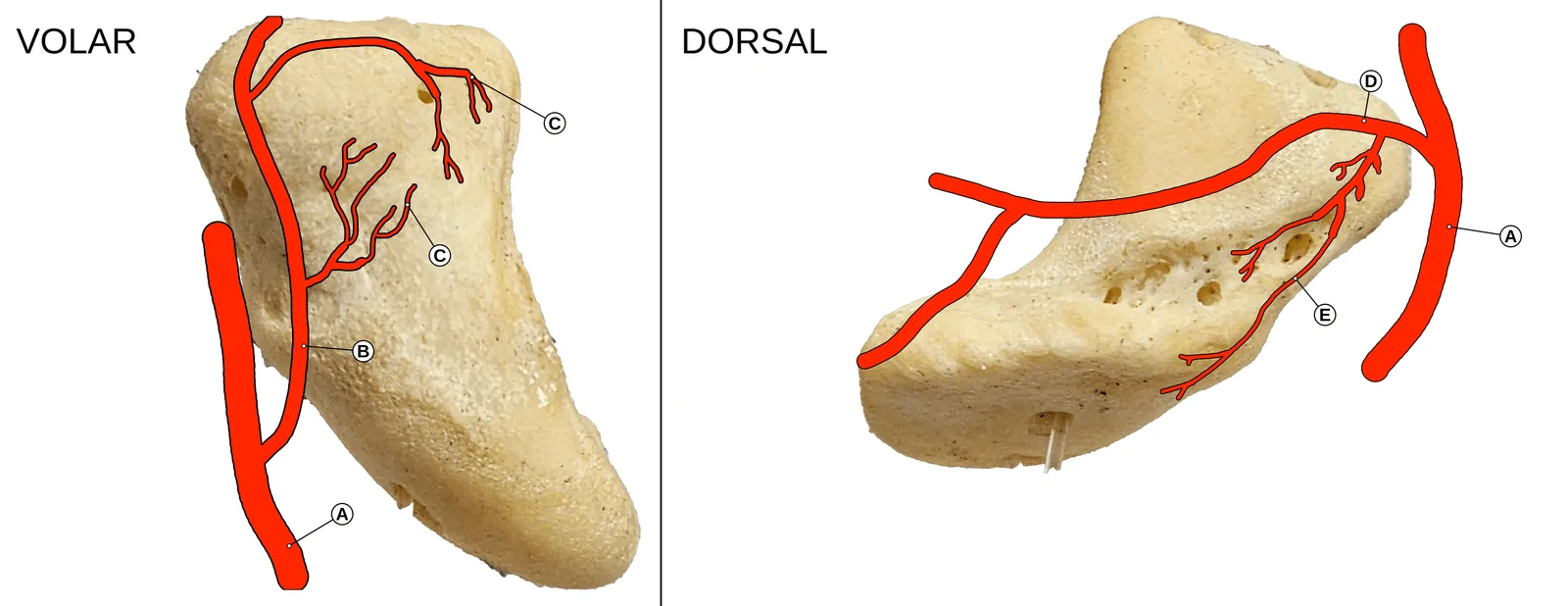
Volar and dorsal views of left scaphoid with arterial supply overlaid A: Radial artery B: Superficial palmar branch of radial artery C: Volar scaphoid branches D: Dorsal carpal branch of radial artery E: Dorsal scaphoid branches These images were created by Rohin Wong specifically for this study using Pixelmator, an iOS application.

Plain radiographs of the wrist with dedicated scaphoid views remain the first-line investigation following clinical assessment, but a proportion of true fractures are not visible on initial films, particularly when non-displaced. Reported rates of occult fracture despite normal initial radiographs range from 7.3% to 23% [11,12].

Following a negative or equivocal initial radiograph, further imaging with CT or MRI is commonly performed to identify occult fractures. Other modalities, including scintigraphy, ultrasound, and tomosynthesis, have also been described but are less widely adopted [13-15]. A 2019 survey of 74 NHS trusts found considerable variation in practice. A total of 38 trusts offered MRI, 25 used CT, and 11 relied on repeat radiography as a follow-up test to a suspected occult scaphoid injury [16].

CT offers rapid access in most centres, reducing unnecessary immobilisation and providing useful detail on fracture angulation and displacement relevant to surgical planning [12,17]. Disadvantages include ionising radiation exposure and an inability to directly assess soft tissue injury or bone vascularity.

MRI avoids ionising radiation and offers superior soft tissue characterisation, including assessment of ligamentous injury, fragment vascularity, and bone marrow oedema. Its disadvantages include limited and variable access, standard contraindications (ferromagnetic implants, pacemakers, severe claustrophobia), and a recognised risk of overdiagnosis and overtreatment of clinically insignificant marrow oedema [18].

A recent interdisciplinary consensus statement identified radiography as the agreed first-line investigation, with CT favoured for assessing osseous consolidation or postoperative appearances, and MRI favoured for evaluating vascularity in suspected non-union [19]. UK NICE guidance currently recommends considering MRI as the first-line follow-up investigation following suspicion of a radiologically occult scaphoid fracture, although uptake of this recommendation across the UK has been reported as limited [20,21].

This review is directly relevant to local and national clinical pathway design. Access to MRI varies considerably between centres, and if CT were shown to offer comparable diagnostic performance in appropriately imaged patients, this could support pathway changes that reduce delays to scanning and the number of sequential scans a patient undergoes.

To our knowledge, existing systematic reviews addressing CT and MRI for occult scaphoid fracture have relied on indirect comparison, pooling accuracy data from separate CT and MRI study cohorts. Therefore, the primary aim of this review was to determine the pooled diagnostic accuracy of CT for detecting occult scaphoid fracture in studies where the same patient cohort underwent both CT and MRI.

## Review

Methods

This systematic review was conducted in accordance with the Preferred Reporting Items for Systematic Reviews and Meta-Analyses (PRISMA) 2020 guidelines [22].

Search Strategy

OVID MEDLINE, OVID Embase, and the Cochrane Library were searched. The search strategy combined the following concepts: Concept 1: (scaphoid* OR "scaphoid bone" OR "scaphoid fracture*" OR "carpal navicular" - combined with exp Scaphoid Bone/) AND Concept 2: (fracture* OR injur* - combined with exp Fractures, Bone/) AND Concept 3: (CT OR "computed tomography" OR "computerised tomography" OR "computerized tomography" - combined with exp Tomography, X-Ray Computed/) AND Concept 4: (MRI OR "magnetic resonance imaging" OR MR - combined with exp Magnetic Resonance Imaging/).

No initial date restriction was applied, and studies published up to August 31, 2026, were eligible for inclusion. Reference lists of included studies and relevant previous systematic reviews were also searched for additional eligible studies.

Following de-duplication, two independent reviewers screened the titles and abstracts for eligibility. Full-text articles were subsequently retrieved and assessed against the inclusion criteria. Any disagreements were discussed with a third reviewer.

Eligibility Criteria

Eligible studies enrolled adults aged 18 years or older with clinical suspicion of scaphoid fracture and a negative or equivocal initial radiograph. Participants were required to subsequently undergo both CT and MRI. A reference standard had to be stated. Studies were required to be published in English with full-text availability. Systematic reviews, case reports, case series (n<10) and cadaveric studies were excluded from this review.

Studies were retained for the systematic review when they met the clinical and imaging eligibility criteria. Studies affected by partial verification or incorporation bias were retained for narrative description and risk-of-bias assessment but excluded from quantitative synthesis.

Outcomes

The primary outcome was the diagnostic accuracy of CT, expressed as sensitivity and specificity for detection of occult scaphoid fracture. The primary quantitative synthesis included studies with extractable paired CT/MRI diagnostic data.

The secondary outcome was concordance between CT and MRI findings, expressed as percentage agreement and Cohen’s kappa in studies using MRI as the reference standard. Where a reference standard independent of both index tests was used, sensitivity and specificity were reported for both CT and MRI.

Data Extraction

Data extraction was completed independently by two reviewers using a standardised Excel spreadsheet. The extracted variables included location, study characteristics, number of patients, imaging modality and reference standard.

For each included study, the total number of recruited patients and the subgroup undergoing both CT and MRI were extracted. Where studies enrolled patients who received only one imaging modality, the paired CT-MRI subgroup was distinguished from the overall study population in Table 1.

The number of scaphoid fractures, sensitivity, and specificity were extracted for the paired CT-MRI subgroup where possible. Where patient-level data permitted construction of a 2 × 2 contingency table, sensitivity and specificity were calculated directly. Where studies reported accuracy for scaphoid fracture alongside other carpal injuries, only scaphoid-specific data were extracted.

Where paired-subgroup-specific data could not be derived from the available text or supplementary material, the sensitivity and specificity reported by the original study authors were used and identified in Table 1. Where no extractable or reported value was available, or where the denominator prevented calculation, the value was recorded as not available.

Risk of Bias and Study Categorisation

Two reviewers independently assessed the included studies using QUADAS-2, with disagreements resolved by a third reviewer [23]. This was used to evaluate areas such as patient selection, index tests, reference standards, and flow and timing.

A pre-specified decision rule was applied to determine how each study's risk of bias affected its inclusion in quantitative synthesis. Studies in which the second index test was performed only in patients with a specific result (positive or negative) on the first index test were considered to have partial verification or incorporation bias. Such studies were retained for narrative description but excluded from all quantitative synthesis, as this design precludes valid estimation of paired diagnostic accuracy. Studies using MRI as the reference standard for CT were retained in the primary pooled CT analysis, since this is a recognised approach where an invasive gold standard is impractical. However, MRI's own sensitivity and specificity could not be validly assessed in these studies, as MRI cannot be discordant with itself. These studies also contributed to a secondary CT-MRI concordance analysis. Studies using a reference standard independent of both index tests contributed accuracy estimates for both CT and MRI.

Statistical Analysis

Pooled CT sensitivity and specificity with 95% confidence intervals were calculated using a bivariate random-effects model based on the Reitsma method and fitted using restricted maximum likelihood in the mada package in R [24]. This approach accounts for variation between study sizes and the correlation between sensitivity and specificity.

Heterogeneity was assessed using I² calculated using multiple approaches, including Zhou and Dendukuri and Holling et al. sample-size-adjusted and unadjusted methods, given the instability of individual I² estimates when few studies contribute to a meta-analysis [25,26].

Concordance between CT and MRI in studies using MRI as the reference standard was assessed using percentage agreement and Cohen’s kappa. Kappa was interpreted using standard benchmarks [27]. A pooled kappa was not calculated because of heterogeneity in study design and the absence of an established approach appropriate to the available data.

Results

Study Selection

The literature search identified 968 records. Following removal of 253 duplicates, 715 records underwent title and abstract screening. Of these, 658 were excluded, and 57 full-text reports were assessed for eligibility. Eight studies met the inclusion criteria and were included in the systematic review.

The included studies were all prospective and were conducted across England, France, Sweden, the Netherlands, Turkey, Australia, and Austria. The total cohort sizes ranged from 20 to 296 patients. One study compared cone-beam CT (CBCT) with MRI, one compared dual-energy CT (DECT) with MRI, one compared multidetector CT (MDCT) with MRI, one compared CT, MRI, and bone scintigraphy, and the remaining studies compared conventional CT with MRI [28-35].

The study selection process is illustrated in the PRISMA flow diagram (Figure 2).

**Figure 2:**
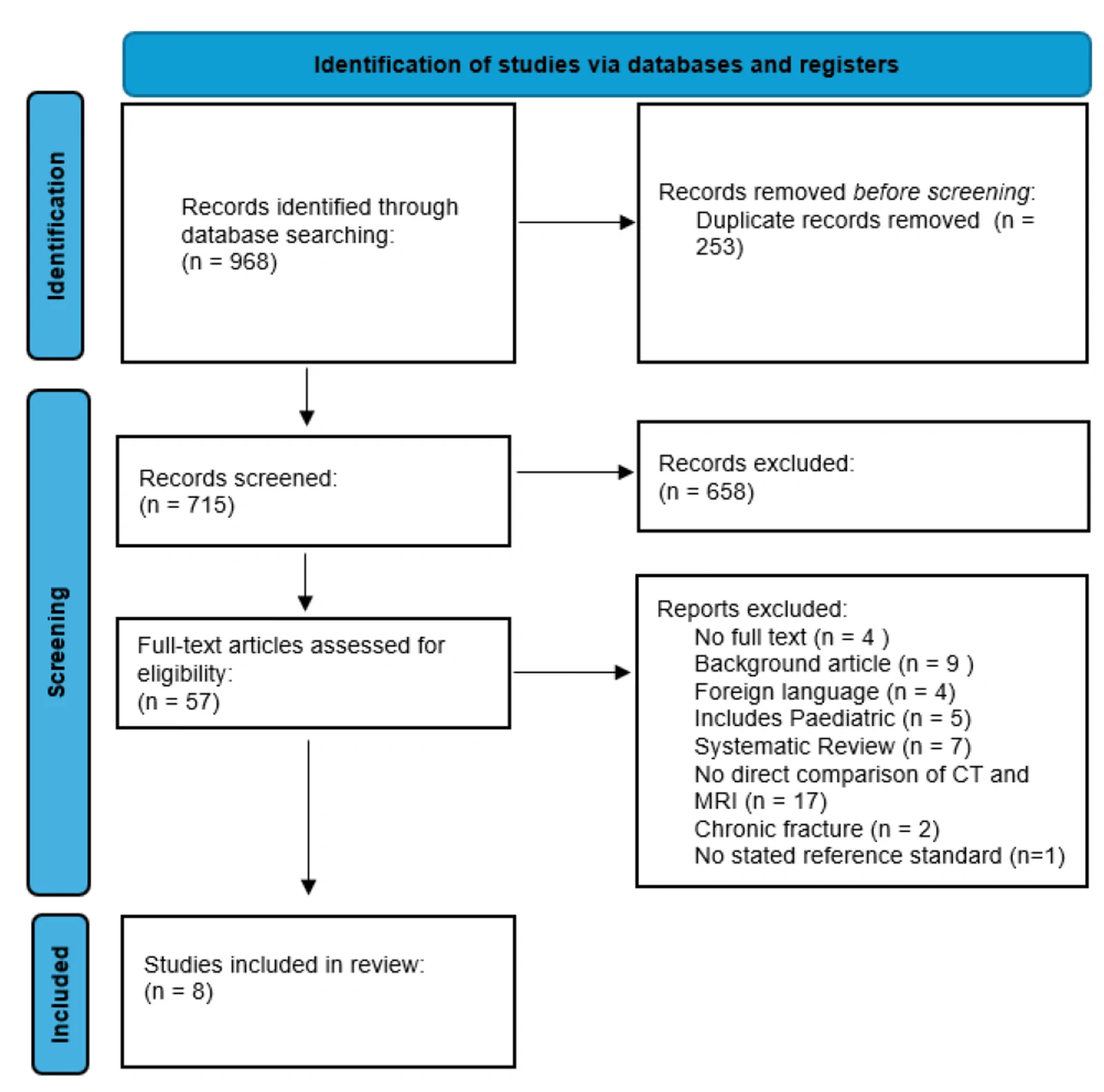
PRISMA 2020 flow diagram Preferred Reporting Items for Systematic Reviews and Meta-Analyses (PRISMA) 2020 [22].

Characteristics of Included Studies

Two studies, Jørgsholm et al. and Cruickshank et al. were excluded from quantitative synthesis because the second index test was performed only in patients with a positive result on the first test, introducing partial verification or incorporation bias [31,34].

The eight included studies and their imaging modalities, reference standards, sample sizes, paired CT-MRI populations, and diagnostic accuracy results are presented in Table 1 below.

**Table 1:** Baseline characteristics of the included studies. ^†^ Excluded from all quantitative synthesis due to partial verification/incorporation bias (second index test performed only in patients positive/negative on the first). *Two independent readers reported. DECT: Dual-energy computed tomography. CBCT: Cone-beam computed tomography, MDCT: Multidetector computed tomography, MRI: Magnetic resonance imaging, XR: X-ray.

Author	Imaging	Reference standard	n	n (CT+MRI)	n (scaphoid fracture)	CT sensitivity	CT specificity	MRI sensitivity	MRI specificity
Xie et al., 2021 [28]	DECT+MRI	MRI	20	20	6	A:100% B:33%^*^	A:64% B:79%^*^	100%	100%
Borel et al., 2017 [29]	CBCT+MRI	MRI	49	49	16	94%	97%	100%	100%
De Zwart et al., 2016 [30]	CT+MRI+BS	Clinical exam + XR	33	33	3	33%	100%	67%	100%
Jorgsholm et al., 2013 [31] ^†^	MRI±CT	MRI	296	122	122	95%	N/A	100%	N/A
Ilica et al., 2011 [32]	MDCT+MRI	MRI	54	54	16	88%	100%	100%	100%
Mallee et al., 2011 [33]	CT+MRI	Repeat XR (6wk)	34	34	6	67%	96%	67%	89%
Cruickshank et al., 2007 [34]^ †^	CT±MRI	Clinical exam + XR + MRI	47	8	2	100%	100%	100%	100%
Memarsadeghi et al., 2006 [35]	CT+MRI	Repeat XR (6wk)	29	29	11	73%	100%	100%	100%

Primary Outcome: Pooled CT Diagnostic Accuracy

Six studies contributed to the primary pooled CT analysis [28-30,32,33,35]. Xie et al. reported findings from two independent CT readers. Reader A was used for the primary analysis because no reader seniority or consensus reading was specified in the original study [28].

Pooled CT sensitivity and specificity, calculated using the bivariate random-effects model, were 79% (95% CI 61.5-89.8%) and 94.4% (95% CI 82.1-98.4%), respectively, using Reader A's data. Overall diagnostic accuracy was high (AUC 0.916). The partial AUC, restricted to the range of false-positive rates observed across the included studies, was lower (0.853), reflecting the narrow spread of false-positive rates in this dataset.

Heterogeneity estimates were reasonably consistent across methods (I² 0-10.6% depending on approach: Zhou and Dendukuri 0%; Holling unadjusted 8.2-10.6%; Holling sample-size adjusted 0.7-0.9%) [25,26]. The bivariate model also estimated a boundary correlation (1.000) between the sensitivity and specificity variance components.

Sensitivity Analysis Using Reader B 

Substituting Reader B's data for Xie et al. yielded a lower pooled CT sensitivity of 69.2% (95% CI 48.0-84.6%), with an unchanged specificity of 94.4% (95% CI 85.5-98.0%) and a similarly high AUC (0.918; partial AUC 0.760) [28]. As with the primary analysis, the model estimated a boundary correlation between variance components (−1.000). Heterogeneity estimates were more inconsistent across methods (I² 0-67.7%: Zhou and Dendukuri 0%; Holling unadjusted 66.5-67.7%; Holling sample-size adjusted 12-14.7%) [25,26].

Secondary Outcome: CT-MRI Concordance

Three studies included in the primary pooled analysis used MRI as the reference standard and provided data suitable for CT-MRI concordance analysis [28,29,33].

Concordance between CT and MRI was almost perfect across two of the three studies (kappa: 0.91) [29,33]. Xie et al. (2021), the one study using dual-energy CT (DECT), showed markedly lower concordance with MRI (kappa 0.519 for Reader A; 0.125 for Reader B, respectively), and agreement between the study's two CT readers was itself only moderate (kappa 0.429) [28]. Inter-reader concordance between Reader A and Reader B for CT fracture detection was calculated as percentage agreement and Cohen's kappa from the study's patient-level supplementary data. The wide range of concordance within a single study, alongside the technical limitations the original authors report, including lower contrast resolution and an inability to suppress cortical bone signal on calcium-subtracted images, suggests that Xie et al.'s concordance findings may reflect DECT-specific interpretive challenges rather than being representative of conventional CT-MRI agreement. Notably, all three contributing studies evaluated CT sub-modalities (CBCT, MDCT, DECT) rather than conventional CT. The concordance results are presented in Table 2.

**Table 2:** Concordance between CT and MRI findings in studies using MRI as the reference standard

Study	n	% Agreement	Cohen's kappa	Interpretation
Xie et al., 2021 [28]	20			
Reader A: CT vs MRI		75.0%	0.519	Moderate
Reader B: CT vs MRI		65.0%	0.125	Slight
Reader A vs Reader B		70.0%	0.429	Moderate
Borel et al., 2017 [29]	49	95.90%	0.91	Almost perfect
Ilica et al., 2011 [32]	54	96.40%	0.91	Almost perfect

Studies Using an Independent Reference Standard

Three studies used a reference standard independent of both CT and MRI. Memarsadeghi et al. reported CT sensitivity and specificity of 73% and 100%, respectively, compared with 100% sensitivity and 100% specificity for MRI [35]. Mallee et al. reported identical CT and MRI sensitivity of 67%. CT specificity was 96%, compared with 89% for MRI [33]. De Zwart et al. reported CT sensitivity of 33% and MRI sensitivity of 67%, while specificity was 100% for both modalities [30]. Because only three studies used an independent reference standard, these results were not formally pooled.

Risk of Bias

Risk of bias was assessed utilising the QUADAS-2 assessment [23]. This demonstrated variable methodological quality across the eight included studies. One study was judged to have low overall risk of bias, and seven had high overall risk.

Most studies were judged to have a low risk of bias in the patient selection and index test domains, reflecting the prospective design of most studies and the use of predefined diagnostic criteria for image interpretation. The most frequent sources of bias were identified within the reference standard and flow and timing domains. Four studies used MRI as the reference standard and were therefore considered to have a low risk of bias in this domain. In contrast, several studies relied on follow-up radiographs and/or clinical assessment as the reference standard, which may fail to identify occult scaphoid fractures and were consequently judged to have a higher risk of bias. Concerns within the flow and timing domain commonly related to loss to follow-up, incomplete verification, and the selective use of advanced imaging in subsets of patients. Partial verification bias was observed in studies where MRI or CT was performed only in selected participants following initial imaging findings. The index test domain consistently demonstrated the lowest risk of bias, as image interpretation was generally undertaken using predefined diagnostic criteria and with blinding to the results of comparator imaging modalities. The QUADAS-2 assessment is presented in Table 3.

**Table 3:** Risk of bias assessed via the QUADAS-2 rating scale QUADAS-2: Quality Assessment of Diagnostic Accuracy Studies 2

Study	Patient Selection	Index Test	Reference Standard	Flow & Timing	Overall Risk of Bias
Xie et al., 2021 [28]	High	Low	Low	High	High
Borel et al., 2017 [29]	Low	Low	Low	Low	Low
de Zwart et al., 2016 [30]	Low	Low	High	Low	High
Jørgsholm et al., 2013 [31]	High	Low	Low	High	High
Ilica et al., 2011 [32]	Low	Low	Low	High	High
Mallee et al., 2011 [33]	Low	Low	High	High	High
Cruickshank et al., 2007 [34]	Low	Low	High	High	High
Memarsadeghi et al., 2006 [35]	Low	Low	High	Low	High

Discussion

This systematic review evaluates the comparative diagnostic accuracy of CT in patients with clinically suspected, radiographically occult scaphoid fractures. Six studies contributed to the pooled CT diagnostic accuracy analysis. Using Reader A data from the study reporting two CT readers, pooled CT sensitivity and specificity were 79% (95% CI 61.5-89.8%) and 94.4% (95% CI 82.1-98.4%), respectively. The sensitivity analysis using Reader B data produced a lower pooled sensitivity of 69.2% (95% CI 48.0-84.6%), while specificity remained the same at 94.4% (95% CI 85.5-98.0%).

The findings of this review highlight that CT and MRI frequently produced similar specificity within the paired cohorts studied. However, the relatively limited sensitivity of CT as well as the uncertainty surrounding the pooled estimates, in addition to the observed variable concordance, indicate that the two modalities are not interchangeable in all clinical circumstances.

Our findings are in keeping with previous systematic reviews that assessed CT and MRI using independent reference standards. They reported CT to have a sensitivity ranging from approximately 72% to 93% and specificity from 96% to 100% [36-39], whereas MRI sensitivity and specificity were in the region of 94.2% and 97.9%, respectively [36-39].

However, when MRI is used as the reference standard for CT, MRI sensitivity and specificity cannot be independently estimated within that study because MRI defines the reference classification. Consequently, these studies provide information about CT performance relative to MRI and CT-MRI concordance rather than an unbiased comparison of the diagnostic accuracy of both modalities. This methodological distinction is important as the three studies using an independent reference standard produced more mixed comparative findings. Memarsadeghi et al. reported equivalent specificity and higher MRI sensitivity, while Mallee et al. reported identical sensitivity with higher CT specificity. De Zwart et al. reported higher MRI sensitivity with equivalent specificity [30,33,35].

Clinical Implications

MRI remains an important investigation for suspected occult scaphoid fractures, both due to high diagnostic accuracy and because it provides information beyond fracture morphology, including assessment of bone marrow, soft tissues, and potentially alternative diagnoses [19]. CT has advantages related to accessibility, speed, assessment of fracture morphology, and evaluation of displacement or angulation. These characteristics make CT useful particularly in preoperative and postoperative assessment [12,17,19]. Developments in abbreviated MRI protocols may also influence future diagnostic pathways by reducing scanning duration and potentially improving MRI accessibility as well as uptake across the UK [40].

Strengths and Limitations

A principal strength of this review was its methodological design, with a focus on studies in which the same patients underwent both CT and MRI. This addresses a limitation of previous indirect comparisons based on separate study populations. The review also distinguished studies according to the independence of their reference standards. This allows the evidence supporting CT accuracy to be interpreted according to the degree to which the reference standard was independent of the index tests.

However, it should be acknowledged that the use of paired-cohort studies with MRI has resulted in the CT pooled estimate being drawn from a smaller and more selected sample size than would be available from an indirect synthesis of the wider CT accuracy literature. Whilst this may limit statistical precision relative to larger indirect reviews, the internal validity remains unaffected. 

Additionally, heterogeneity, which was high particularly in the calculation for accuracy with Reader B, could be represented by the variation in imaging protocols such as CT slice thickness, MRI field strength, sequence protocols and inclusion of CBCT/MDCT/DECT variants, reference standard interval and population characteristics. These were not formally explored via subgroup analysis or meta-regression given the limited number of studies. Concordance (kappa) could not be formally pooled across studies given the absence of an established method for combining kappa estimates from heterogeneous designs; findings were therefore reported study-by-study rather than a single summary figure. 

The bivariate random-effects model estimated a boundary correlation of 1.00 for the pooled calculation using Reader A and -1.00 using Reader B between the sensitivity and specificity variance components. Given the limited number of contributing studies, this likely restricted the model’s ability to reliably partition between-study variation; these values indicate instability in estimation of the between-study covariance structure, rather than evidence of a true perfect correlation between sensitivity and specificity.

Future Research

Future primary studies could further investigate direct comparisons of CT and MRI for occult scaphoid fractures. These should apply a single reference standard, independent of both index tests, uniformly to all participants regardless of either test's result. Furthermore, larger, harmonised-protocol studies comparing CT sub-modalities (CBCT, MDCT, DECT) against an independent reference standard such as MRI would also allow meaningful sub-modality analysis, helping to identify whether any specific modality would be able to confer, in particular, a comparable sensitivity to MRI.

## Conclusions

For adults presenting with clinical suspicion of scaphoid fractures and negative initial X-rays, existing recommendations, including NICE guidelines, favour MRI as the second-line investigation. In this systematic review, CT demonstrated high specificity but more limited sensitivity for occult scaphoid fractures across six paired CT-MRI studies. CT-MRI concordance was almost perfect in two of the three studies, although findings were less consistent in the DECT study. In agreement with previous systematic review literature, these findings suggest that MRI should be favoured for diagnostic accuracy in occult scaphoid fractures; however, CT has a role in selected patients, particularly where MRI is unavailable or contraindicated, or for surgical planning. However, the limited number of studies using a reference standard independent of both CT and MRI, together with heterogeneity in imaging protocols and reference standards, limits the certainty with which CT and MRI can be considered diagnostically equivalent. Further prospective studies using a uniform reference standard independent of both index tests are warranted.
